# Determination of the optimal matching position for setup images and minimal setup margins in adjuvant radiotherapy of breast and lymph nodes treated in voluntary deep inhalation breath-hold

**DOI:** 10.1186/s13014-015-0383-y

**Published:** 2015-04-03

**Authors:** Marko Laaksomaa, Mika Kapanen, Mikko Haltamo, Tanja Skyttä, Seppo Peltola, Simo Hyödynmaa, Pirkko-Liisa Kellokumpu-Lehtinen

**Affiliations:** Department of Oncology, PO BOX 2000 (Teiskontie 35), Tampere University Hospital (TAUH), FI-33521 Tampere, Finland; Department of Medical Physics, Tampere University Hospital (TAUH), PO BOX 2000, Teiskontie 35, FI-33521 Tampere, Finland; School of Medicine, University of Tampere, PO BOX 607, FI-33101 Tampere, Finland

**Keywords:** Radiotherapy, Breast cancer, Setup errors, Image guidance, Breath-hold

## Abstract

**Background:**

Adjuvant radiotherapy (RT) of left-sided breast cancer is increasingly performed in voluntary deep inspiration breath-hold (vDIBH). The aim of this study was to estimate the reproducibility of breath-hold level (BHL) and to find optimal bony landmarks for matching of orthogonal setup images to minimise setup margins.

**Methods:**

1067 sets of images with an orthogonal setup and tangential field from 67 patients were retrospectively analysed. Residual position errors were determined in the tangential treatment field images for different matches of the setup images. Variation of patient posture and BHL were analysed for position errors of the vertebrae, clavicula, ribs and sternum in the setup and tangential field images. The BHL was controlled with a Varian RPM® system. Setup margins were calculated using the van Herk’s formula. Patients who underwent lymph node irradiation were also investigated.

**Results:**

For the breast alone, the midway compromise of the ribs and sternum was the best general choice for matching of the setup images. The required margins were 6.5 mm and 5.3 mm in superior-inferior (SI) and lateral/anterior-posterior (LAT/AP) directions, respectively. With the individually optimised image matching position also including the vertebrae, slightly smaller margins of 6.0 mm and 4.8 mm were achieved, respectively. With the individually optimised match, margins of 7.5 mm and 10.8 mm should be used in LAT and SI directions, respectively, for the lymph node regions. These margins were considered too large. The reproducibility of the BHL was within 5 mm in the AP direction for 75% of patients.

**Conclusions:**

The smallest setup margins were obtained when the matching position of the setup images was individually optimised for each patient. Optimal match for the breast alone is not optimal for the lymph node region, and, therefore, a threshold of 5 mm was introduced for residual position errors of the sternum, upper vertebrae, clavicula and chest wall to retain minimal setup margins of 5 mm. Because random interfraction variation in patient posture was large, we recommend daily online image guidance. The BHL should be verified with image guidance.

## Background

The voluntary deep inhalation breath-hold technique (vDIBH) facilitates sparing of the heart and ipsilateral lung during radiotherapy (RT) of left-sided breast cancer [1,2]. The efficacy of this technique has been known for a long time [3] but the unavailability of resources has limited its use in many clinics. Normal vDIBH treatment takes no longer than 20 minutes, which is an average time for several other treatments [4]. Together with modern RT equipment, use of vDIBH for the left-sided breast cancer RT is increasing and becoming a part of daily practice in RT centres.

With the free breathing technique, image guidance for position verification is performed at the random respiratory phase, but average movement of the chest wall during normal free breathing is only 2 mm [5]. With a spirometer-based DIBH technique, median intra- and interfraction position variations of 2.0 mm and 3.6 mm, respectively, have been observed for passive markers on the breast surface in the anterior-posterior direction [6]. Relatively large movements have also occurred especially in the superior-inferior direction having medians of 2.4 mm and 4.3 mm, respectively [6]. The variation is due to physiological and human reasons and may cause uncertainty in patient position with respect to treatment beams. In previous studies the reproducibility of breath-hold level (BHL) has been measured using several techniques, such as infrared markers [6], surface imaging [7], cine images [8] or orthogonal planar images [1].

Because of the uncertainties described above, the setup margins required for the breast tissue and lymph node regions have to be determined for DIBH techniques. In addition, due to non-rigid variation in patient anatomy, selection of appropriate bony landmarks for the matching of the setup images is not obvious. There are no recommendations on matching of the setup images and on verification of the BHL. To the best of our knowledge, all the factors above together with interobserver variation in image matching have not been previously investigated in the literature with consistent patient material collected throughout the entire course of treatment. The effect of variation in the breath-hold pattern on the position accuracy of the landmarks used for the image matching has not been properly addressed in the literature.

The main goal of this study was to find optimal bony landmarks for matching of the orthogonal setup images in RT of left-sided breast cancer treated in vDIBH. The optimality of each landmark was evaluated based on residual position errors in the images. Especially, residual errors in tangential treatment field images were investigated to maximally spare the heart and ipsilateral lung but position errors of the lymph node regions were also determined. Variation in the BHL was investigated throughout the entire course of treatment. Motivation for evaluation of the optimal matching position was given by showing position errors caused by nonrigid patient anatomy and variation in breath-hold patterns. We routinely use vDIBH treatments for all left-sided breast cancer patients younger than 70 years capable for breath holding of 20 s.

## Methods and materials

### Patient group, respiratory gating protocol and planning CT acquisition

**Figure 1:**
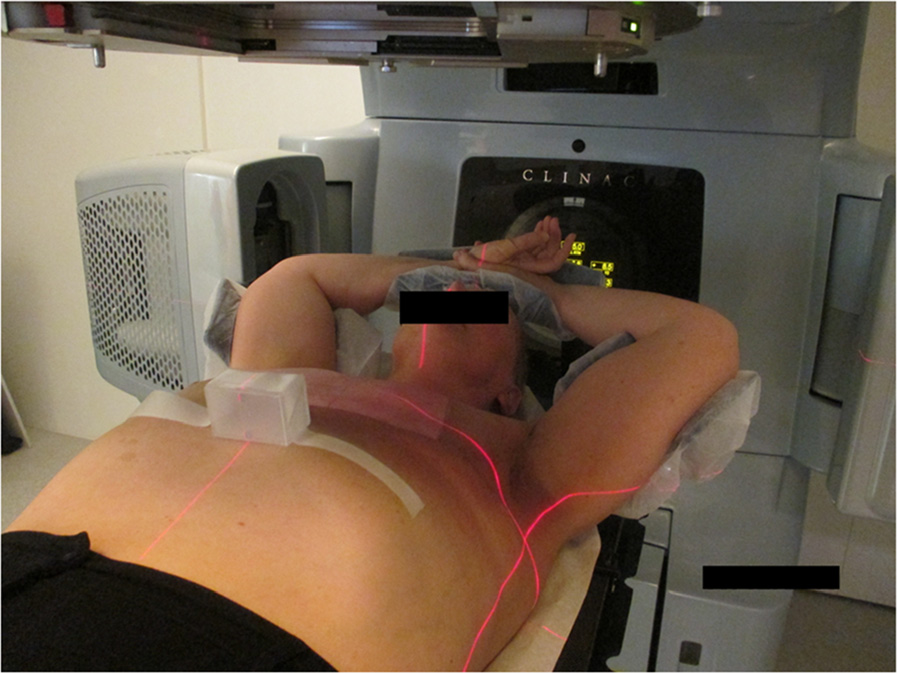
**Patient fixation.** The breast board is tilted by 5° and it has two arm support devices. The patient lies supine with both arms lifted above the head. The RPM marker block is taped to patient skin. The laser lines are aligned to three tattoo marks placed in free breathing on patient skin: two marks on both lateral sides and one on the sternum. There is also one mark on the abdomen to minimize patient rotation. After the laser alignment, translational shifts are performed to the treatment isocenter before the image guidance procedure.

**Figure 2:**
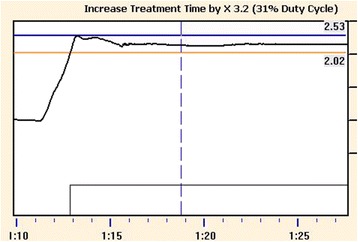
**Breath-hold curve and gating window.** Breath-hold curve (black curve) tends to stabilize within a few seconds (typically 2 seconds). The CT imaging and treatment delivery were performed during a stable breath-hold period defined by upper and lower limits (blue and orange vertical lines, respectively). In this case the limits of ± 2.5 mm were chosen around the (average) reference breath-hold level based on several successive breath-holds. The numbers on the right show the location of the gating window with respect to baseline level (maximal exhale in free breathing).

A total of 67 consecutive left-sided breast cancer patients receiving adjuvant RT following breast conserving surgery were retrospectively investigated using offline analysis. Of these, 23 patients were treated for axillary and supraclavicular regions due to lymph node involvement. The mean patient age was 55 years. Patient fixation was carried out with Candor’s ConBine fixation device (Candor, Gislev, Denmark) (Figure 1). A Varian RPM respiratory gating system, version 1.7 (Varian Medical Systems, Palo Alto, CA, USA) was used for respiratory monitoring. An RPM block with two dot markers was placed between the xyphoid process and the umbilicus (4.5 cm below the xyphoid on average, as previously reported [1,9]) to detect maximal anterior-posterior respiratory movement. Each patient was carefully informed about the breath-hold procedure. Before the planning CT, patients were audio coached to perform reproducible and stable breath-holds for periods of 12–15 s at least 4–5 times and one breath-hold exceeding 20 s. If these conditions were met, a gating window from ±2 to ±5 mm was set around the average BHL. The window width was chosen based on the upper and lower limits defined by the majority of the breath-holds (Figure 2). Audio guidance was given to reach the average BHL (reference level) for the acquisition of the treatment planning CT. When necessary, the reference level was corrected to correspond to the average situation during the imaging. The breath-hold curve and gating window in the CT scanning were recorded and used as references for treatment delivery. The CT imaging was done with vDIBH technique at 120 kVp using either a Philips Brilliance Big Bore (Philips Medical Systems, Eindhoven, The Netherlands) or a Toshiba Aquilion LB (Toshiba Medical System, Tokyo, Japan) scanner and a slice thickness of 3 mm. This study was performed in compliance with the principles of good clinical practice, the Helsinki Declaration, and federal and institutional guidelines of Tampere University Hospital. Permission for use of the patient data was obtained from the institution (R12536). The study had no effect on the treatment of the patients.

### Treatment protocol, image guidance protocol and image matching tools

Patients were treated to 50 Gy at 25 fractions or to 42.56 Gy at 16 fractions with two opposing tangential fields for the breast volume (n = 44) and with two additional oblique anterior fields and one posterior field for simultaneous lymph node irradiation (n = 23) with a 6 MV photon beam of the Clinac 2300 iX accelerator (Varian Medical Systems). All the beams were planned to the same isocenter. The orthogonal kV-images were acquired daily in breath-hold with an onboard imaging system (OBI) at 75 kV/200 mA/25 ms for the anterior images and at 95 kV/200 mA/200 ms for the lateral images (with spatial resolution of 0.13 mm with detector distance of 150 cm). Online images were analysed in treatment console (4D Console 11.1) using image blending and image overlay tools. Patient setup corrections were based on these images. An action level of 0 mm was used for couch corrections and tangential portal field images (MV) were acquired after the couch corrections to document residual position errors (with spatial resolution of 0.26 mm with detector distance of 150 cm). A time delay of few seconds (typically 2 s) was used before the setup image acquisition and treatment delivery to reach stable breath-hold pattern close to reference level. BHL stability was maximised with audio guidance given by the radiation therapists. The radiation therapists used also an offline image matching tool (Offline Review in Aria 11, Varian Medical Systems) in conjunction with the online image guidance to find optimal individual matching position for each patient. Sessions having orthogonal image pair and tangential field image were retrospectively analysed by one observer (ML) using the offline tool. Altogether, 1067 online image sets were analysed.

### Selection of bony landmarks for matching of the orthogonal setup images and evaluation of patient posture

The combined position errors caused by nonrigid patient anatomy, patient rotation and variation in the breath-hold pattern were retrospectively investigated. This was done by determining variations in the distances between the relevant landmarks. The landmarks used to most explicitly measure patient rotation were the upper vertebrae (UPPER_V) and lower vertebrae (LOWER_V). The rotation was measured as translational shifts of these landmarks. Position errors between the middle part of the vertebrae (MID_V) and the upper or lower part of the sternum (UPPER_ST or LOWER_ST) were used to estimate the reproducibility of the BHL.

**Figure 3:**
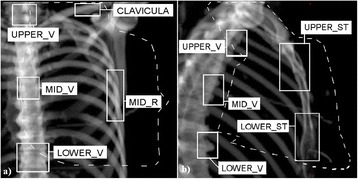
**Selection of the bony landmarks relevant for breast cancer RT.** The bony landmarks (open white boxes) chosen in **a)** anterior reference image and **b)** lateral reference image. The landmarks were chosen at the presented levels with respect to the target and the humeral head. Notice that the UPPER_V and MID_V are not at the same level in **a)** and **b)**. The distances between the landmarks were within ± 2 cm for all the patients enabling the comparison of patient rotation. The projection of the target covering the whole breast and lymph node region is illustrated with a dashed line.

Three bony landmarks relevant for the target volume were used for matching of the orthogonal setup images in the online situation as well as in the retrospective analysis. The landmarks were the middle part of the ribs (MID_R), the MID_V and the UPPER_ST. The LOWER_ST was not applied for the matching because position corrections in superior-inferior direction can be determined much more accurately from the UPPER_ST and corrections in anterior-posterior direction can also be determined from the UPPER_ST. The latter is possible because the UPPER_ST region extended caudally to the middle of the sternum and to the middle of the target volume of the whole breast. The bony landmarks assumed to correlate with the axillary and supraclavicular lymph node regions were the upper vertebrae and clavicula. All the bony landmarks are presented in Figure 3.

### Residual position errors after the online match

Based on our previous clinical experience, the orthogonal setup images were matched online to the midway compromise of the sternum and the ribs. Residual position errors of the landmarks demonstrated in Figure 3 were retrospectively measured. Because the setup images were matched online by thirty experienced radiation therapists, the residual errors obtained include interobserver variation in image matching. These errors were used to calculate setup margins with the van Herk’s formula (*m* = 2.5*Σ* + 0.7*σ*) [10], where Σ is the systematic error (standard deviation of patient mean errors) and σ is the random error (root-mean-square over all deviations around patient mean errors).

All directions are given in the orthogonal setup images as superior-inferior (SI), anterior-posterior or vertical (AP) and lateral (LAT). The AP and LAT directions are combined (to 1D) in the tangential images (denoted as AP/LAT).

### Residual position errors in tangential field images and evaluation of optimal bony landmarks for matching of the setup images

Residual position errors in the tangential images were retrospectively determined for the chest wall and breast surface after the online matching of the orthogonal setup images and couch corrections. For the chest wall, the error in the AP/LAT direction was defined from the error of central lung distance (CLD) and in the SI direction from the displacement of the whole visible chest wall structure in that direction. The breast surface in the reference image was defined from the projection of body contour drawn in the CT image while the breast surface was clearly visible in the online image. For the breast surface, the errors in the AP/LAT and SI directions were determined from the central flash distance (CFD) and inferior central margin (ICM), respectively [11]. Potential swelling and daily displacements of the breast are included in these measures. Position errors between the laser setup and the treatment position were also determined for the chest wall and breast surface in the tangential images.

The orthogonal setup images were retrospectively and separatively matched to each of the three bony landmarks described above to find the optimal matching position resulting in minimal residual position errors in the tangential images. Also “midway compromises” of these structures were investigated. This means that the match was set exactly at the midpoint of the two structures in both setup images. In the SI direction, the average of the two setup images was chosen. These image alignments were performed only to the indicated landmark or landmarks ignoring the other landmarks. Residual errors of the landmarks were determined from the matched orthogonal images. Because the residual position errors in the tangential image were known for the online match of the orthogonal images (after couch correction), it is possible to recalculate the residual position errors in the tangential image when the matching of the orthogonal images is altered. This was done by converting the changed readings in the LAT and AP directions into the AP/LAT direction using the angle of the tangential field and trigonometrical functions.

### Statistical analysis

Statistical tests were applied to evaluate whether the position errors are significantly different. Because the systematic errors were normally distributed, the two-tailed F-test was used for the systematic errors (test for equality of variances). Because the random errors are not normally distributed, the non-parametric Wilcoxon rank sum test was used for the random errors (test for equality of means). A *p*-value ≤ 0.05 was considered statistically significant.

## Results

### Variations in patient posture and distances between the landmarks

Table 1 demonstrates the combined position errors caused by patient rotation, non-rigid anatomy and variation in breath-hold pattern. Systematic and random position errors of the UPPER_V and LOWER_V indicating rotation of the vertebrae were equal to or below 2.2 mm in all directions. Variations in breath-hold patterns had only slight effect on the distance between the vertebrae and ribs in LAT direction because position errors between them were below 1.5 mm. The largest effect existed in the SI direction, where both systematic and random position errors were large in the distances between all the investigated landmarks, and was most pronounced between the UPPER_ST and MID_V.

**Table 1:** **Residual errors (mm) of the distances between the landmarks caused by variations in breath-hold level and non-rigid patient anatomy**

**Landmarks and the investigated images**	**Clinical importance**	**Systematic error Σ**			**Random error σ**		
		**AP**	**SI**	**LAT**	**AP**	**SI**	**LAT**
UPPER_ST-MID_V	Breath-hold level and rotation about LAT axis	3.4	5.0	-	2.5	3.3	-
(LAT image)							
MID_V-MID_R	Breath-hold movements and rotation about AP axis	-	3.6	1.3	-	3.0	1.3
(AP image)							
MID_R-UPPER_ST	Deformation caused by breath-hold	-	3.8	-	-	2.6	-
(AP+LAT image)							
UPPER_V-LOWER_V	Rotation about AP and LAT axes	1.4	0.8	2.2	1.4	1.2	1.9
(AP+LAT image)							
UPPER_ST-LOWER_ST	Non-rigidity and rotation of the sternum	1.7	0.9	-	1.3	1.1	-

### Reproducibility of the BHL

The realised average BHL window width was 5.9 mm (range from 4 to 10 mm). The widest acceptable window width of 10 mm was applied only for patients with the poorest breath-hold capability (n = 2). Interfraction reproducibility of the mean RPM level was −0.7 ± 3.3 mm (mean shift ± SD). The constancy of the RPM signal during the breath-hold was 2.0 ± 2.3 mm (mean shift ± SD).

Systematic error of the distance between the UPPER_ST and MID_V was 5.0 mm (1SD) in the SI direction and 3.4 mm (1SD) in the AP direction. The signed average, average of absolute values and median of absolute values of the errors were 2.2, 4.3 and 3.9 mm, respectively in the SI direction, and 0.8, 2.6 and 1.6 mm, respectively in the AP direction. The large systematic errors (given as deviations) were caused by large systematic position errors of the sternum exceeding 8 mm (thickness of the sternum) observed for few patients in the SI direction (n = 8), in the AP direction (n = 1) or in both directions (n = 4). Random displacements between these landmarks were smaller: 3.3 mm (1SD) in the SI direction and 2.5 mm (1SD) in the AP direction. For the LOWER_ST the corresponding errors were only 0.3 mm larger.

We investigated the effect of the marker block location on the reproducibility of the BHL with two groups of patients: 5 patients with largest systematic BHL errors and 5 patients with smallest systematic BHL errors. However, the marker block was placed similarly for these patient groups (on average 4.5 and 4.3 cm caudally from the xyphoid, respectively), indicating no correlation between the systematic BHL error and the location of the marker block.

### Best bony landmarks for alignment of the orthogonal setup images

Table 2 presents residual errors in the tangential field images after the orthogonal setup images were matched to different landmarks. The averages of the signed patient mean errors were below 1 mm. The best general matching position was the compromise of the sternum and the ribs (UPPER_ST + MID_R, *p* ≤ 0.02) to minimise the residual position errors in the tangential images. The sternum alone was the best landmark for 34 % of the cases in the SI direction. The poorest image matching location was the vertebrae alone (*p* ≤ 0.03). The vertebrae were the best landmark in the SI direction in only 5% of the cases, whereas the corresponding number for the ribs and the compromise matches were between 9–14 %. Including the vertebrae in the combination of landmarks increased the setup margin at least by 1.9 mm in the AP/LAT direction and 0.8 mm in the SI direction. For patients with the largest position errors caused by nonrigid anatomy (n = 22), the margins were increased by 2.4 mm and 1.7 mm, respectively. The random errors were not significantly different (*p* ≥ 0.12) for all the landmarks in the LAT/AP direction. The systematic errors were not significantly different (*p* ≥ 0.14) in the SI direction for all other landmarks, except for the MID_V and MID_V+MID_R (*p* ≤ 0.01).

**Table 2:** **The residual errors (mm) of the chest wall and the breast surface (in parenthesis) after different alignments of the orthogonal kV setup images**

	**Systematic error Σ**		**Random error σ**		**Margins in mm**	
**Objects**	**AP/LAT direction**	**SI direction**	**AP/LAT direction**	**SI direction**	**AP/LAT direction**	**SI direction**
Laser setup	3.5 (4.3)	4.5 (5.1)	3.6 (3.8)	4.5 (4.5)	11.3 (13.4)	14.4 (15.9)
MID_V	2.9 (3.4)	4.2 (5.5)	2.3 (2.6)	3.5 (4.1)	8.9 (10.3)	13.0 (16.6)
MID_R+UPPER_ST	1.5 (2.6)	1.9 (3.2)	2.2 (2.4)	2.6 (3.0)	5.3 (8.2)	6.6 (10.1)
MID_V+UPPER_ST	1.8 (2.9)	2.3 (3.9)	2.1 (2.4)	2.7 (3.3)	6.0 (8.9)	7.6 (12.1)
MID_V+MID_R	2.9 (3.4)	3.1 (4.4)	2.2 (2.5)	2.8 (3.3)	8.8 (10.3)	9.7 (13.3)
MID_V+UPPER_ST+MID_R	2.2 (3.1)	2.2 (3.7)	2.4 (2.3)	2.6 (3.0)	7.2 (9.4)	7.3 (11.4)
UPPER_ST	-	2.3 (3.5)	-	2.9 (3.3)	-	7.8 (11.1)
MID_R	-	2.8 (3.8)	-	3.0 (3.3)	-	9.1 (11.8)
Online match to optimal position^1^	1.3 (2.6)	1.8 (3.1)	2.1 (2.4)	2.2 (3.1)	4.8 (8.2)	6.0 (9.9)

^1^includes interobserver variation in image matching.

The best match in Table 2 was the online match (*p* ≤ 0.02). In this match, the matching location was evaluated individually for each patient during the treatment course using offline image matching in conjunction with the online image guided RT (IGRT). Table 3 presents residual errors after individually optimised matching. The residual errors in Table 3 demonstrate that in the online match the sternum was weighted more than the ribs in the SI direction and much more than the vertebrae in both the SI and LAT/AP directions. For patients with the largest systematic BHL errors, the residual errors in the tangential images were slightly greater after the most optimal match than that obtained for the other patients, but the rank of the optimal matching positions remained the same. The AP image alone was not sufficient to determine localisation in the SI direction in most cases, because the setup margins after the MID_R+MID_V match (best alignment in the AP image) was 3.2 mm larger for the chest wall than with the best compromise match of the AP+LAT setup images together.

**Table 3:** **Residual errors (mm) of different landmarks after the daily online match**

	**Systematic error Σ**			**Random error σ**		
**Object**	**AP**	**SI**	**LAT**	**AP**	**SI**	**LAT**
MID_V^1^	3.2	4.1	1.4	2.4	3.4	1.4
UPPER_ST^1^	1.1	1.6	-	1.5	2.3	-
MID_R^1^	-	2.8	0.7	-	3.0	1.2
Setup corrections^1^	3.4	4.1	2.4	3.3	4.4	2.7

^1^includes interobserver variation in image matching.

### Best bony landmarks for setup image alignment when lymph nodes are also irradiated

The residual errors of the clavicula and upper vertebrae are presented for different matching positions in Table 4. Obviously, the best matching location for the lymph node volume was the vertebrae alone, but this would lead to remarkable errors in tangential field images ruining the benefits of the vDIBH technique. The compromise of the MID_V+UPPER_ST+MID_R would lead to an overall margin of 7 mm which would still compromise any benefit. In the optimal match for the whole breast alone (MID_R+UPPER_ST and in the online match) the margin in the SI direction would be approximately 1 cm.

**Table 4:** **Residual errors (mm) of the upper vertebrae and clavicula (in parenthesis) when the orthogonal setup images are matched to different positions**

	**Systematic error Σ**		**Random error σ**		**Margins in mm**	
**Matching position**	**LAT direction**	**SI direction**	**LAT direction**	**SI direction**	**LAT direction**	**SI direction**
MID_V	1.3 (2.5)	1.1 (2.3)	1.3 (2.2)	1.8 (3.1)	4.2 (7.8)	4.0 (7.9)
MID_R+UPPER _ST	1.9 (2.3)	3.0 (2.3)	1.3 (2.1)	2.3 (2.2)	5.7 (7.2)	9.1 (7.3)
MID_V+UPPER_ST	1.3 (2.5)	2.5 (2.4)	1.3 (2.2)	1.8 (2.4)	4.2 (7.8)	7.5 (7.7)
MID_V+MID_R	1.5 (2.3)	1.4 (2.0)	1.2 (2.0)	1.5 (2.3)	4.6 (7.2)	4.6 (6.6)
MID_V+UPPER_ST+MID_R	1.5 (2.3)	2.2 (2.0)	1.2 (2.0)	1.9 (2.2)	4.6 (7.2)	6.8 (6.5)
UPPER_ST	-	4.5 (3.4)	-	3.0 (3.0)	-	13.4 (10.6)
MID_R	-	2.4 (2.3)	-	2.4 (2.3)	-	7.7 (7.4)
Online match to optimal position^1^	1.7 (2.4)	3.5 (2.9)	1.4 (2.2)	2.9 (2.9)	5.3 (7.5)	10.8 (9.2)

^1^includes interobserver variation in image matching.

## Discussion

In this study, the optimality of bony landmarks was investigated for matching of the orthogonal setup images in order to obtain the smallest residual errors in tangential treatment field images in RT of left-sided breast cancer treated with the vDIBH technique. Because the patient anatomy is nonrigid and there are variations in the breath-hold level, we considered it important to find best landmarks for matching of the setup images. The margins obtained were determined for daily IGRT. Residual errors after the online match in treatment situations and the required margins were also investigated for the lymph node areas. The residual position errors were determined for the breast tissue from tangential portal images.

It has been reported that portal images underestimate position errors, especially in the superior-inferior direction (by up to 50 %) when compared to cone beam CT (CBCT) [12]. However, we considered that this potential limitation can be eliminated by expanding the setup margins correspondingly for the breast tissue in the SI direction. It should be noticed that the separation of residual errors in anterior-posterior and lateral directions is not possible from tangential images. This is not a limitation when tangential (or almost tangential) beams are used to treat the breast volume. The discrepancy between MV and kV imaging isocentres were confirmed to be within 1 mm for the used directions and thus it has a negligible effect.

### Reproducibility of the BHL

An average BHL window of 5.9 mm was achieved in this study. This value allows variation of ±3 mm around the reference level defined in the CT imaging (Figure 2). The result is consistent with median inter-fraction variability of 4.9 mm obtained with spirometer-based monitoring for the same marker position on the patient abdomen [6]. The choice of the maximal window width (10 mm in this study) is always a compromise of the acceptable treatment time and the poorest breath-hold capability among the eligible patients. It seems that the appropriate window width also depends on the location of the marker block on patient body [6]. A relatively wide window of 10 mm has been used, when the block has been placed on patient’s abdominal surface [1], while windows as narrow as 2–3 mm have been used, when the block is placed on the chest wall and the visual breath-hold guidance has been given [13]. A literature review and visits to other RT units indicate that these two locations are used to place the marker block in DIBH treatments of the breast cancer. In our study, the marker block was placed between the xyphoid and umbilicus, as recommended in some recent studies [1,9,14] and by the RPM manufacturer.

With the presented protocol, average of the absolute BHL errors, the average of the signed errors and median of the absolute errors were consistent within −2.0 to +0.5 mm (current result – literature value) with the recent literature values obtained with several techniques, such as spirometric monitoring [6], visual coaching [7], cine imaging [8] and fluoroscopic investigation of residual errors [2]. McIntosh et al. have evaluated BHL reproducibility by investigating displacements between the spine and sternum obtained from lateral images (RPM system, no reported coaching, *n* = 10) [1]. The reported mean error of 1 mm (range 0–3 mm) is equal to our current results, but the slightly larger deviation of the BHL errors in our study may be due to different criteria of the eligible patients. The SD of the RPM signal variations, however, was 1 mm smaller in the present study. Although different techniques set some limitations for direct comparison of the current results with those reported in the literature, similarity of the results suggests that the current results may have wide importance.

The retrospective analysis of the lateral setup images showed that the BHL has systematically changed for some patients after the planning CT. The BHL shifts seem to be patient-related because the reproducibility of the RPM systems and inter-system differences were within 1 mm in our phantom studies. A patient may move the chest wall in a different way to reach the breath-hold in the CT imaging and treatment situation. This may be caused by the time gap between the planning imaging and beginning of treatment. In the majority of patients, the displacement errors between the sternum and vertebrae (indicating the BHL) were largest in the SI direction (signed average 2.2 mm). For all patients, the maximal errors were still much smaller than the height of the BHL (average height 2.8 cm). Most (90%) of the BHL changes were detected already in the first three treatment fractions. This indicates that it is possible to detect and potentially correct the systematic BHL errors already after 3–5 first treatment fractions based on the lateral setup images [15]. In some cases, the (uncorrected) lower BHL may mean that slightly higher doses are delivered to the heart than planned, even though the setup images were precisely aligned based on the chest wall and breast and residual errors in tangential image are minimal.

The current results imply that more breathing training may be useful for patients with poor breath-hold capability before the treatment planning CT and treatment. Chopra et al. [16] have reported some benefits of breathing training, resulting in increased breath-hold time and tidal volume but variation of the breast position was modestly reduced. Visual coaching has also been suggested to improve the DIBH reproducibility [7]. We consider that further studies have to be carried out to find effective methods for correction of residual position errors in DIBH treatments.

It can be assumed that contribution of interfraction BHL errors is eliminated for each single landmark when it is used alone to match the images; this contribution exists in the compromise matches and in the distances between the landmarks. Because all the images used for the IGRT (anterior and lateral setup images, and tangential field image) were acquired during different breath-hold cycles, it can be assumed that residual errors of the landmarks in the directions that can be determined from at least two images include contributions from intra-fractional BHL errors.

### Best bony landmarks for alignment of the setup images

#### Whole breast alone

In our previous study, we have discovered that the compromise of the MID_R+UPPER_ST+MID_V is the best general choice for matching of the orthogonal setup images in RT of the whole breast performed in FB [17]. It should be remembered that all the compromise matches in this study are “midway compromises” (as defined in Methods and Materials) unless otherwise stated. Ranking of the setup image matching locations seems nearly identical for the FB and vDIBH patient groups, but the residual errors are larger for vDIBH patient group and the use of the vertebrae in the image matching increases margins more than for the patients treated in FB. This is due to variation in breath-hold patterns within the vDIBH patient group. Because of the reasons stated above, the online match was the best match for the vDIBH group. In this match, the matching location was determined individually for each patient. For the patients with good BHL reproducibility, the compromise of the MID_R+UPPER_ST+MID_V (obtained for the FB group) was generally considered as the best choice, while for the patients with poor BHL reproducibility the compromise of the MID_R+UPPER_ST was generally the best solution. Therefore, it is most optimal to determine the weighting of the vertebrae individually. This requires careful investigation of the setup and tangential images acquired in first few treatment fractions. A new matching location should be found for the setup images when residual systematic error of the chest wall exceeds 3.5 mm in the AP/LAT direction measured from the tangential image (the margin of 5.0 mm remains sufficient by accounting for random errors). The average matching location obtained for the patient group was closer to the sternum than to the vertebrae as seen from the residual errors presented in Table 3.

We recommend that daily image guidance is applied because large inter-fractional setup variation was observed as demonstrated in Table 3; this recommendation is consistent with the literature [18,19]. Bartlett et al. [4] have investigated the setup errors related to vDIBH with CBCT and tangential field images. The couch corrections between the laser setup and CBCT alignment were nearly identical in our findings (Σ and σ of setup errors within 0.6 mm in both studies).

#### Lymph node irradiation

Hjelstuen et al. have demonstrated that the DIBH technique can be used to reduce OAR irradiation while maintaining appropriate coverage of the whole breast and axillary-, supraclavicular- and internal mammary lymph node regions [20]. Stranzl et al. have shown the benefit of the DIBH technique for the irradiation of the internal mammary lymph nodes [21]. In the current study the most cranially located bony landmarks were visible in kV images and the lymph node regions were assumed to correlate with the structures c7-Th1 and clavicula presented in Figure 3.

In our previous study with the FB technique, we concluded that the compromise of the MID_R+UPPER_ST+MID_V was the best general choice for the setup image matching, because setup margins of 5 mm were obtained for the lymph node region and chest wall [17]. However, due to variation in the BHL pattern within the patient group, use of the vertebrae in the image matching increases the residual errors in the tangential field images mostly in the AP/LAT direction, which is not acceptable, because the dose to the heart and lung increases. Application of the optimal setup image matching position obtained for the breast (and use of minimal margins for the breast) would lead to suboptimal setup margins of even 1.1 cm for the upper vertebrae in SI direction. This means that the pursued margins of 5 mm should be doubled. The best general matching location for the patients with lymph node irradiation would be the compromise of the MID_R+UPPER_ST+MID_V in the SI direction and the UPPER_ST+MID_R in the AP/LAT direction. This kind of match, however, is prone to error in daily practice and does not alone guarantee optimal coverage of the whole PTV for individual patients.

Based on the results in given Tables 2 and 4 we concluded that the best general compromise for the setup image matching would be the compromise of the UPPER_ST+MID_R for the vDIBH patient group having lymph node irradiation. In this case, a threshold should be applied for the residual position errors of the landmarks representing the lymph node regions. In practise, application of the threshold means that some kind of compromise of the MID_R+UPPER_ST+MID_V is used for matching of the setup images when the lymph nodes are irradiated (not necessarily “midway compromise” for the MID_V). Obviously it is most optimal to evaluate appropriate weighting of the vertebrae individually for each patient to obtain the best compromise match. This may be needed even in every treatment session. Daily IGRT protocol should be used to ensure that the residual position errors of the sternum, vertebrae, clavicula and chest wall do not exceed the threshold of 5 mm after the treatment isocenter has been selected in order to achieve margins of 5 mm sufficient for the lymph node regions and the chest wall (critical for heart and lung). Usually, the SI direction is not critical for the breast volume because quite large margins can be applied safely. For the lymph nodes, the AP direction is not critical when conformal anterior-posterior treatment fields are used. However, residual error of 5 mm should not be exceeded for the chest wall in the AP/LAT direction. If the threshold is exceeded for any critical landmark, BHL and/or patient posture should be corrected and patient re-imaged before treatment delivery.

Offline image inspection was found as an effective method to detect and reduce systematic errors in conjunction with the online IGRT [22]. To obtain the full benefits of the IGRT, we systematically determine the best setup image alignment for each individual patient and investigate position errors of the landmarks during the first 3–5 fractions using both online and offline image inspection.

## Conclusions

The best matching location for the orthogonal setup images was the compromise of the UPPER_ST+MID_R for RT of the whole breast treated in vDIBH. This resulted in minimal residual position errors and setup margins in the tangential field images. The rounded setup margins were 6 mm in the SI direction and 5 mm in the AP/LAT direction. By selecting the image matching location individually for each patient, 0.5 mm smaller margins were achieved. In 25 % of the fractions, the BHL differed at least by 5 mm in the AP direction and/or 8 mm in the SI direction from the planned values when measured from the lateral setup images. Verification of the BHL using the image guidance is recommended.

For patients with lymph node involvement, use of the optimal match obtained for the breast volume is recommended, but a threshold of 5 mm is proposed for residual position errors of the upper vertebrae, clavicula, sternum and chest wall. When this threshold is exceeded, corrective actions should be considered in order to confirm the planned dose to the heart, lung, breast, lymph node region and larynx. We conclude that both the online and offline image inspection are needed to find the best matching location for individual patients and to verify the BHL reproducibility already in the beginning of the treatment course. Daily online image guidance is recommended because random interfractional setup variation ranged maximally to 4.4 mm (1 SD).

## Consent

Oral informed consent was obtained from the patient for the publication of the patient data in this report and any accompanying images.
